# Caffeine Interaction with Glutamate Receptor Gene GRIN2A: Parkinson's Disease in Swedish Population

**DOI:** 10.1371/journal.pone.0099294

**Published:** 2014-06-10

**Authors:** Naomi Yamada-Fowler, Mats Fredrikson, Peter Söderkvist

**Affiliations:** 1 Division of Cell Biology, Department of Clinical and Experimental Medicine, Linköping University, Linköping, Sweden; 2 Division of Occupational and Environmental Medicine, Department of Clinical and Experimental Medicine, Faculty of Health Sciences, Linköping University, Linköping, Sweden; Mathematical Institute, Hungary

## Abstract

A complex interplay between genetic and environmental factors is thought to be involved in the etiology of Parkinson's disease (PD). A recent genome-wide association and interaction study (GWAIS) identified *GRIN2A*, which encodes an NMDA-glutamate-receptor subunit involved in brain's excitatory neurotransmission, as a PD genetic modifier in inverse association with caffeine intake. Here in, we attempted to replicate the reported association of a single nucleotide polymorphism, *GRIN2A*_rs4998386, and its interaction with caffeine intake with PD in patient-control study in an ethnically homogenous population in southeastern Sweden, as consistent and independent genetic association studies are the gold standard for the validation of genome-wide association studies. All the subjects (193 sporadic PD patients and 377 controls) were genotyped, and the caffeine intake data was obtained by questionnaire. We observed an association between rs4998386 and PD with odds ratio (OR) of 0.61, 95% confidence intervals (CI) of 0.39–0.96, p = 0.03, under a model excluding rare TT allele. There was also a strong significance in joint effects of gene and caffeine on PD risk (TC heavy caffeine vs. CC light caffeine: OR = 0.38, 95%CI = [0.20–0.70], p = 0.002) and gene-caffeine interaction (OR = 0.998, 95%CI = [0.991–0.999], p<0.001). Overall, our results are in support of the findings of the GWAIS and provided additional evidence indicating PD protective effects of coffee drinking/caffeine intake as well as the interaction with glutamate receptor genotypes.

## Introduction

Recent advances in genetic research in familial form of Parkinson's disease (PD) have identified several causative genes and shed lights on underlying disease mechanisms. However, in vast majority (>85%) of PD is sporadic form and only limited genetic risk factors have been identified thus far. Sporadic form of PD is thought to be a complex disorder caused by combinations of multiple genetic and environmental factors. It is likely that specific variants of a gene cannot alone explain disease status in a given individual, but instead, they confer an increased risk. Genome wide association studies (GWAS) are capable of capturing such weak genetic effects and was hoped to become a powerful tool to identify susceptibility loci of PD. However, despite the expectation, over 20 GWAS related studies, including original GWAS, re-analysis, and meta-analysis, conducted on PD population to date have identified only a few candidate genes. Among them, the most consistently replicated main effects detected are for *SNCA* and *MAPT*, but others are less consistent [1], [2].

Lately, Hamza et al [3] proposed a new post-GWAS approach called genome-wide association and interaction study (GWAIS). This approach is based on the concept that inclusion of environmental factors can help identify genes that are missed in GWAS. They examined each SNP's main-effect on PD risk plus its interaction with a well-established PD life-style factor, coffee/caffeine intake, using the data on 1,458 persons with PD and 931 without PD from the NeuroGenetics Research Consortium (NGRC) in the Discovery stage. The GWAIS reviled strong PD association and caffeine interaction with rs4998386 and the neighboring single nucleotide polymorphisms (SNP) in *GRIN2A*, which prompted further analysis using additional three datasets consist of 2472 cases and 2848 controls (the total of four cohorts). Among heavy coffee-drinkers, rs4998386_T (minor allele) carriers had lower PD risk than rs4998386_CC carriers with odds ratio (OR) of 0.51, p  =  7×10^−8^ (TC to CC excluding rare heterogeneous TT genotype). Not surprisingly, *GRINA2A* encodes an NMDA-glutamate-receptor subunit involved in brain's excitatory neurotransmission, and both caffeine-like adenosine antagonists and *GRIN2A*-related glutamate antagonists are being tested in PD clinical trials.

Consistent and independent genetic association studies still remain to be the gold standard for the GWAS validation. Thus, here in, we attempt to replicate the previously reported association of rs4998386 and its interaction with the PD protective effects of coffee/caffeine intake in patient-control study in an ethnically homogenous population in southeastern Sweden.

## Methodology

### Ethics

The study subjects signed informed consent, and the study was approved by the Ethics Committee at the Faculty of Health Sciences, Linköping University, Sweden, Dnr. 00–253.

### Study population

Blood samples were collected from 193 L-dopa positive PD patients (121 male, 72 female) age 49-85 years, in southeast Sweden, who were visiting the Clinic from Geriatrics and Neurology, University Hospital in Lingköping, Sweden. The control group contains 377 unrelated Swedish individuals (189 male, 188 female) age 50–87 years old randomly collected from the normal population in southeast Sweden, the same study base as patients. Patient and control groups have been frequency matched for both age and gender.

### DNA extraction

Genomic DNA was isolated from peripheral blood leukocytes with QIAamp DNA Blood Maxi Kit (VWR, Stockholm, Sweden).

### Real-time PCR allelic discrimination assays

Real-time PCR allelic discrimination assays were conducted using TaqMan SNP Genotyping Assays (Applied Biosystems, Foster City, CA). The assay (TaqMan SNP Genotyping Assays ID: C_28018721_20) perform genotyping of *GRIN2A*_rs4998386 (C→T). Genotypes were assessed by the TaqMan allele-specific assay method using the 7900HT Fast Real-Time PCR System according to the manufacturer's protocols (Applied Biosystems), and scored by the allelic discrimination program of Sequence Detection System (SDS).

### Assessment of caffeine intake

Coffee intake data was obtained by a questionnaire consists of lifestyle/environmental exposure questions. The daily caffeine intake was deducted from the cups-per-day coffee consumption reported by the participants. There was no question about other sources of caffeine (tea, soda or caffeine-containing medications). Generally in Sweden, major source of caffeine intake is coffee especially for the age group of the study subjects, and decaffeinated coffee is not commonly consumed [4]. To classify light or heavy caffeine intake, cut-off point of 237.8 mg/day was used for the comparison with Hamza's data. The value is one of the cohorts' median total daily caffeine intake in their study.

### Statistical evaluation

Chi-square analysis and Fisher's exact tests were performed to compare the genotype and the allele frequencies of *GRIN2A*_rs4998386 in PD patients and the control group. Gene-Environment interactions were analyzed with logistic regression model. All statistical analyses were performed using Stata 12 statistical analysis software (StataCorp LP, College Station, TX). Statistical significance was defined at P values less than 0.05.

## Results

We examined the association of *GRIN2A*_rs4998386 with PD within the southeast Sweden cohort (Table 1). The genotype frequencies of the PD patients and control were in Hardy-Weinberg equilibrium (p>0.05). The PD protectiveness in T carrier was in agreement with the previous report by Hamza et al [3]. Under a dominant model (CC vs. TC+TT), there was a near significant difference in the genotype frequency between PD and control groups (OR = 0.65, 95%CI = [0.42–1.01], p = 0.06), and under a comparison between TC to CC, i.e. excluding rare TT genotype as Hamza's study [3] indicated that TT is rare and the frequency varied significantly across the datasets, a significant difference was observed (OR = 0.61, 95%CI = [0.39–0.96], p = 0.03) between PD patients and control, in which TC genotype was indicative of being protective. Likewise, allele frequency excluding TT genotype was also significantly different between PD and control (OR = 0.64, 95%CI = [0.42–0.99], p = 0.04.)

**Table 1:** Genotype and allele frequencies for GRIN2A_rs4998386 in Parkinson's disease case/control in southeastern Swedish population.

		Genotype counts (%)					Allele frequency (%)			
	N	CC	TC	TT	OR(95% CI)	p^a^	C	T	OR(95% CI)	p^a^
PD	193	159 (82.0)	30 (16.0)	4 (2.0)	0.65(0.42–1.01)^c^	0.06^c^	348 (90.1)	38^b^ (9.9)	0.73(0.49–1.09)^b^	0.12^b^
	-	-	-	-	0.61(0.39–0.96)^d^	0.03^d^	348 (92.0)	30^d^ (8.0)	0.64(0.42–0.99)^d^	0.04^d^ ^d^ ^d^
Control	377	284 (75.3)	88 (23.3)	5 (1.3)	-	-	656 (87.0)	98^b^ (13.0)	-	-
	-	-	-	-	-	-	656 (88.2)	88^d^ (11.8)	-	-

a P-value: Chi-square test.

b Including rare TT genotype.

c Dominant model. Wild type CC vs. T carrier (TC+TT).

d Excluding rare TT genotype.

We then analyzed the effects of caffeine intake and *GRIN2A*_rs4998386 on PD (Table 2). Following the Hamza et al's classification [3] based on the median caffeine consumption of PAGE (Parkinson's, Genes, and Environment from the prospective NIH-AARP Diet and Health Study cohort), we classified below or above 237.8 mg/day as either light- or heavy- caffeine consumers, respectively. Interestingly, when rare heterogeneous TT genotype was excluded from the analysis, among the light caffeine consumers, there was a near significance that TC is more PD protective than CC (OR = 0.42, 95%CI = [0.17–1.03], p = 0.06), while in the heavy caffeine consumers, this trend was weaker (OR = 0.69, 95%CI = [0.40–1.17], p = 0.16). The results showed that the coffee dose had somewhat different effects from the data of Hamza et al, in which there were no protective effects of TC in the light coffee drinkers while a large impact existed in the heavy coffee drinking with TC carrier.

**Table 2:** Caffeine intake with GRIN2A_rs4998386 genotype in Parkinson disease case/control in southeastern Swedish population.

	PD/Control	N	CC (%)	TC (%)	TT (%)	OR (95% CI)	p-value
Light caffeine intake^a^	PD patient	58	49 (48.5)	8 (13.8)	1 (1.7)	0.47 (0.21–1.13)^c^	0.09^c^
	Control	75	54 (72.0)	21 (28.0)	0	0.42 (0.17–1.03)^d^	0.06^d^
Heavy caffeine intake^b^	PD patient	135	110 (80.9)	22 (16.2)	3 (2.9)	0.73 (0.44–1.21)^c^	0.21^c^
	Control	302	230 (76.1)	67 (22.2)	5 (1.7)	0.69 (0.40–1.17)^d^	0.16^d^

a Light caffeine intake <237.8 mg/day.

b Heavy caffeine intake >237.8 mg/day.

c Dominant model. Wild type CC vs. T carrier (TC+TT).

d Excluding rare TT genotype.

We also analyzed the impact of caffeine intake alone on PD regardless of the genotype (Table 3). PD protectiveness of caffeine intake was significant with OR = 0.58, 95%CI = 0.39–0.86, p = 0.007.

**Table 3:** Caffeine intake regardless of genotype in Parkinson disease case/control in southeastern Swedish population.

PD/Control	N	Light caffeine intake^a^	Heavy caffeine intake^b^	OR (95% CI)	p-value
PD patient (%)	193	58 (30.0)	135 (70.0)	0.58 (0.39–0.86)	0.007
Control (%)	377	75 (19.9)	302 (80.1)	-	-

a Light caffeine intake <237.8 mg/day.

b Heavy caffeine intake >237.8 mg/day.

Given the genotype and caffeine effects found in our cohort, we investigated joint effects of *GRIN2A*_rs4998386 genotypes and caffeine intake (Table 4). Compared to the light caffeine consumer with *GRIN2A*-rs4998386_CC genotype (the group with highest risk), heavy caffeine intake with CC genotype reduced risk by 47% (OR = 0.53, 95%CI = [0.34–0.83], p = 0.005), having *GRIN2A*_rs4998386_T allele with light caffeine intake shows trend of protectiveness but not statistical significance (OR = 0.42, 95%CI = [0.17–1.03], p = 0.06), and the combination of heavy caffeine intake and *GRIN2A*-rs4998386_TC genotype was associated with a 64% risk reduction (OR = 0.38, 95%CI = [0.20–0.70], p = 0.002). There was also a strongly significant *GRIN2A*-rs4998386 genotype*Caffeine interaction (OR = 0.998, 95%CI = [0.991–0.999], p<0.001).

**Table 4:** PD risk with GRIN2A genotype and caffeine intake.

	Genotype	Caffeine intake	PD	Control	OR (95% CI)	p-value
**Joint effects** a	CC	Light^d^	49	54	reference	-
	CC	Heavy^e^	110	230	0.53 (0.34–0.83)	0.005
	TC	Light d	8	21	0.42 (0.17–1.03)	0.06
	TC	Heavy^e^	22	67	0.38 (0.20–0.70)	0.002
	TT	Light d	1	0	-	-
	TT	Heavy^e^	3	5	0.66 (0.15–2.91)	0.58
**Interaction** b	-	-	193	377	0.998 (0.991–0.999)	<0.001
**Dose-dependent** c	CC	≤200 mg/day	46	37	reference	-
		200–≤400 mg/day	98	189	(0.25–0.69)	<0.001
		400–≤600 mg/day	7	28	(0.08–0.51)	<0.001
		>600 mg/day	8	30	(0.09–0.52)	<0.001
	TC	≤200 mg/day	7	16	reference	-
		200–≤400 mg/day	21	56	(0.31–2.38)	0.76
		400–≤600 mg/day	2	8	(0.10–3.40)	0.69^f^
		>600 mg/day	0	8	-	0.15^f^

a Joint effects of GRIN2A rs4998386 and caffeine intake.

b GRIN2A rs4998386*caffeine interaction.

c Genotype specific dose-dependent effect of caffeine.

d Light caffeine intake <237.8 mg/day.

e Heavy caffeine intake >237.8 mg/day.

f Fisher's exact test. Chi-square is calculated only if all expected cell frequencies are equal to or greater than 5.

Genotype specific dose-dependent effects of caffeine were analyzed (Table 4). The caffeine intake were classified into 4 levels; 0–≤200 mg/day, 200–≤400 mg/day, 400–≤600 mg/day, and >600 mg/day. This classification method was chosen because our caffeine dose data was distributed into several clusters and quartile ranking was not feasible. There was a caffeine dose-dependent PD protectiveness in CC genotype. Caffeine intake of 200–≤400 mg/day was associated with 58% risk reduction (OR = 0.42, 95%CI = [0.25–0.69], p<0.001), and that of 400 mg–≤600 mg/day was associated with a highly significant 80% reduction (OR = 0.20, 95%CI = [0.08–0.51], p<0.001), and >600 mg/day had also strong 79% reduction (OR = 0.21, 95%CI = [0.09–0.52], p<0.001) comparing to the lowest caffeine intake group with 0–≤200 mg/day. However, such caffeine dose effects were not found in the TC genotype.

Additionally, we analyzed caffeine-PD associations with stage of life (age) in our case-control study population as caffeine is often linked with long-term habitual consumption. The population was divided at the median age of 69 years [younger (49–69 years old), older (70–87 years old)]. In the healthy control, the younger group's caffeine consumption was higher than that of the older group [mean caffeine intake (± standard deviation) mg/day; younger 321.1 (±145.3), older 294.2 (±128.5), p = 0.01 (Student's t-test)]. However, there was no such age-group difference in PD population whose caffeine intake was low regardless of the age [mean caffeine intake (± standard deviation) mg/day; younger 276.8 (±134.4), older 271.3 (±125.8)].

## Discussion

The results from a series of GWAS added our understanding of the genetic basis of PD by identifying several loci with common genetic variants associated with PD risk. However, the variants identified by GWAS are often non-causal variants and the mechanisms in which associated loci confer PD risk are yet unclear. Moreover, GWAS do not take gene-environment or gene-lifestyle effects into account to predict the risk [5]. To overcome the weaknesses of GWAS, Hamza el al [3] recently proposed a Post-GWAS analysis called GWAIS (genome-wide association and interaction study). The study utilized the NeuroGenetics Reserch Consortium (NGRC) GWAS as a discovery dataset, and three independent datasets for replication genotyping; PEG (Parkinson, Environment, and GENE), PAGE (Parkinson's, Genes, and Environment form the prospective NIH-AARP Diet and Health Study cohort), and HIHG (Hussman Institute for Human Genomics). They investigated gene-environment interaction in PD, namely, caffeinated-coffee intake and *GRIN2A*, the gene encodes for a subunit of the NMDA-glutamate-receptor, and it was found that carriers of *GRIN2A*_rs4998386-T allele had a lower risk of PD than carriers of rs4998386-CC genotype among heavy coffee drinkers. While the NMDA-receptor is involved in synaptic plasticity and learning, its excessive activation results in excitotoxicity leading to neuronal damage, which is thought to be a mechanism underlies several neurologic or neurodegenerative disorders. Thus, not surprisingly, genetic variants in *GRIN2* have been associated with diseases such as Huntington disease [6], Epilepci [7], ADHD [8], Schizophrenia [9], and bipolar disorder [10]. To regulate NMDA-mediated cellular calcium influx, there is a complex interplay between dopamine receptor D2R and adenosine receptor A_2A_ [11], [12], and the dysregulation can result in neurodegeneration. This may explain neuruo-protective property of caffeine, an adenosine A_2A_ receptor antagonist, as well as the biological mechanism underlying PD risk reduction with coffee intake which has been suggested by several association studies [13].

In this study, we intended to replicate the association of PD risk and *GRIN2A*_rs4998386 genotypes with caffeine intake in a relatively homogenous population in southeastern Sweden. We found an association between *GRIN2A*_rs4998386 genotypes with PD (OR = 0.63, p = 0,04), which is in accordance with Hamza et al. Likewise, coffee intake irrespective of genotype showed significant PD risk reduction with heavy caffeine intake (OR = 0.58, p = 0.009). In terms of the joint effects of *GRIN2A*_rs4998386 with caffeine intake as well as the gene-caffeine interaction, they both were also strongly pronounced in our cohort (CC-light caffeine vs. TC-heavy caffeine: OR = 0.38, p = 0.002 and gene-caffeine interaction: OR = 0.998, P<0.001, respectively). Interestingly, caffeine's dose dependent effects were only found in the CC genotype but not in the TC genotype. This can be seen that TC genotype alone is already protective with 47% risk reduction regardless of the caffeine dose, and increasing caffeine dose does not have any further additive effects, while PD susceptible CC genotype carriers can get benefit from increasing amount of caffeine intake for PD risk reduction by 58% with 200–≤400 mg/day, by 80% with 400–≤600 mg/day, and by 79% with >600 mg/day, respectively. Overall, our data in the southeastern Sweden population is in support of the findings of the GWAIS that *GRIN2A*_rs4998386 TC genotype is PD protective and caffeine intake is jointly enhancing the effects. However, in the GWAIS results, *GRIN2A*_rs4998386-T protectiveness only existed in the higher coffee intake but not in the lower intake group, which is somewhat different from our results. In our study, there was a tendency that the protective effects of *GRIN2A*_rs4998386-T allele were seemingly existed in the population with lower caffeine consumption, but not in the higher caffeine consumers.

One possible explanation for the discordance in terms of the interaction between caffeine/coffee dose and gene is the variations in the coffee intake data between the studies. In GWAIS data [3], four datasets have various coffee (caffeine) intake estimations, and the median value for each data was used as cut-off point to classify light or heavy coffee (caffeine) intake. We analyzed the data based on the caffeine intake deducted from the daily coffee intake and used the median to classify the dose. In our cohort, the median caffeine intake from coffee alone was 380 mg/day, which is 1.6-fold higher than the median of 237.8 mg/day in the PAGE cohort [3], and it could be even higher if other caffeine containing food intake data such as soda and tea were available and included for calculations. Nonetheless, we used cut-off point of 237.8 mg/day for caffeine dose classification for the sake of comparison with Hamza's study. In Sweden, similarly to other Nordic countries, coffee per capita consumption is remarkably high in international comparison, where per capita consumption of roasted coffee in 2004 was approximately 9 kg [14]. For the reference, per capita roasted coffee consumption in the same year in Ireland, France and the Netherlands was only 3.3, 4.4, and 7.1 kg, respectively. It should also be noted that factors such as the types of coffee used, dosages, volumes of cups, and preparation methods can introduce significant variability in coffee/caffeine intake data, and these factors often vary with countries or cultural/ethnical backgrounds. Although accurate estimation of caffeine intake is difficult, it is very important for describing dose-response relationship particularly when gene-environment interactions are studied.

Another point to be considered is the population-specific heterogeneity in PD [15], i.e. the genetic variants associated with PD susceptibility have differential effect on PD risk in various ethnic groups. For example, it is known that there are ethnic variations in *MAPT*. Caucasians display two distinctive haplotypes; H1 and H2, a 900-kb inversion polymorphism on chromosome 17 that contains several genes including *MAPT* [16]. In the population with European ancestry, the H2 haplotype has a 20% prevalence whereas it is nearly absent in East Asians [17]. Even among European populations, *MAPT* ethnic variations were found [16]. While there was a significant association of the H1/H1 genotype and PD in the Serbian population, the same study failed to find such association in a German population. Population-specific heterogeneity in PD risk loci was also found in *LRRK2* variant G2019S, which accounts for 1% of sporadic PD in Caucasians, has a higher frequency in Southern Europe compared to Northern European countries [18]. Similar variations in subgroups of Caucasian populations were found in HLA-DRB5 SNP, rs3129882 [19], where the frequency of risk allele is lower in Northern Europeans than that in Southern Europeans. Furthermore, the effect sizes of PD risk variants may vary in populations of different ancestry. For instance, Sharma et al found that odds ratio of BST1 polymorphism in Asian population was significantly larger than that in Caucasian population [15]. The different magnitude of effect size in populations, in turn, may affect susceptibility to develop PD, age of onset and/or responsiveness to various environmental factors. Therefore in PD association studies, it is important to consider the population-specific genetic heterogeneity in combination with environmental/lifestyle factors in the study population. Populations with same ancestry share not only genetic background, but also tend to have similar lifestyle including diet, which in turn, could add more complexity in gene-environment interactions in PD. In that regard, study cohort with relatively homogeneous ethnic background such as the one from our southeast Swedish population can provide relevant information.

In the previous prospective cohort studies, caffeine intake of one's mid-life was associated with the future development of Alzheimer's disease (AD) [20], dementia [20] and/or the associated parameter(s) [20], [21]. Given the AD-PD similarities, the question arises is whether high caffeine consumption in the earlier stage of life has protective effects on the future disease development in PD as well. Although our case-control design does not allow such analysis directly, our results depicted a clear contrast between the healthy control and the PD populations in terms of the age-group dependent caffeine intake patterns, i.e. the high caffeine intakes in the younger controls, versus, the low caffeine intakes regardless of the age in the PD patients. It would be interesting to see the associations between PD and long-term habitual caffeine intakes in prospective studies.

Taken together, our results support the GWAIS findings of Hamza et al, i.e. the PD protectiveness of the lifestyle factor coffee/caffeine intake and its interaction with a genetic factor *GRIN2A*_rs4998386-T allele, although interaction between the caffeine dose and genotype was characterized somewhat differently between the studies. Given the variability in genetic, lifestyle, and environmental factors even among the subgroups within the Caucasian population, replication studies in various ethnic groups may be needed to further specify the effects of *GRIN2A* genotypes and its caffeine responsiveness on PD protection.
